# Burden of non-communicable diseases in Tunisia, 1990-2017: results from the global burden of disease study

**DOI:** 10.11604/pamj.2021.40.62.30980

**Published:** 2021-09-28

**Authors:** Houyem Khiari, Rym Mallekh, Ines Cherif, Mohamed Hsairi

**Affiliations:** 1 Faculty of Medicine of Tunis, University of Tunis El Manar, Tunis, Tunisia

**Keywords:** Burden, non-communicable diseases, trend, Tunisia

## Abstract

**Introduction:**

non-communicable diseases (NCDs) are the leading cause of mortality and disability worldwide especially in developing countries such as Tunisia. We aimed to describe the national burden of non-communicable diseases in 2017 and to analyze disability-adjusted life year trends from 1990 to 2017 in Tunisia by cause and gender.

**Methods:**

we used Joinpoint regression analysis to assess trends of the age standardized disability-adjusted life year rate from 1990 to 2017 and to determine average annual percentage change.

**Results:**

non-communicable diseases accounted for 87.7% of total disability-adjusted life year in Tunisia in 2017. The five leading causes of this rate in Tunisia in 2017 were cardiovascular diseases, musculoskeletal disorders, neoplasms, mental disorders and neurological disorders. The trend of disability-adjusted life year rate of non-communicable diseases decreased significantly from 23403.2 per 100.000 (95% CI: 20830.2-26285.8) in 1990 to 18454.6 (95% CI: 15611.3-21555.4) in 2017, with a change of -0.9%; p=0.00. The decrease of the age standardized disability-adjusted life year rate concerned mainly cardiovascular diseases and neoplasms secondly. This decrease was more important in female (change=-1.1, p=0.00) in comparison to males (change=-0.7, p=0.00). On the other hand, the increase of the standardized disability-adjusted life year rate was related to musculoskeletal disorders, diabetes, kidney disorders and substance use disorders with a significant annual percentage change of 0.1%, 0.2% and 1.3% (p=0.00) respectively. **Conclusion:** the implementation of the national strategy is the key solution to mitigate the impact of non-communicable diseases in Tunisia.

## Introduction

Non-communicable diseases (NCDs) are the leading cause of mortality and disability worldwide [1]. According to the World Health Organization (WHO), NCDs were responsible for 41 million deaths in the world, which is equal to 71% of all deaths in 2016, including 15 million people aged between 30 and 69 years [2-4]. The burden of NCDs is huge and is still rising worldwide [5,6]. However, the highest risks of dying from these diseases were observed in low and middle-income countries (LMIC) [7,8].

Disease burden of NCDs can be measured by different indicators such as the disability-adjusted life years (DALYs) which is the equivalent of losing one year in good health because of premature death or disease or disability [1]. In Tunisia, NCDs constitute also a major public health issue. According to the WHO, in 2016, NCDs were responsible for 86% of all causes of death (49% from cardiovascular diseases, 12% from cancer, 5% from chronic respiratory diseases and 5% from diabetes) and 16% of premature deaths between 30-70 years [9].

The global epidemic of NCDs was recognized by the United Nations (UN). Therefore, multisectoral national policies and plans were established, framed in the development of the Sustainable Development Goals (SDGs) for the prevention and control of NCDs to be achieved by 2030 [7]. Heads of state and government committed to develop national responses including to reduce by one third premature mortality from NCDs, to strengthen responses to reduce the harmful use of alcohol, to achieve universal health coverage (UHC), to strengthen the implementation of the WHO Framework Convention on Tobacco Control (FCTC), to support the research and development of vaccines and medicines for NCDs that primarily affect developing countries and to provide access to affordable essential medicines and vaccines for NCDs [10].

The WHO global NCDs action plan, implemented collectively between 2013 and 2020 in order to support national efforts by an international cooperation, follows on from commitments made by the United Nation (UN) political declaration on the prevention and control of NCDs. This action plan sets priorities, provides strategic guidance on how countries can apply the roadmap of commitments of the UN and contributes to progress on 9 global NCDs targets to be attained in 2025, including a 25% relative reduction in premature mortality from NCDs by 2025 [11]. In line with the WHO action plan, Tunisia carried out a national strategy for the prevention and control of NCDs for the 2018-2025 period [11]. Understanding global trends and changes in the leading causes of disease burden over time is crucial to assess the achievement of the national strategy targeted goals especially to reduce by 25% the premature mortality from NCDs by 2025 [12]. However, data on mortality are scant in Tunisia.

In this way, the present study aimed to describe the national burden of NCDs in 2017 and to analyze DALYs trends from 1990 to 2017, in Tunisia by cause and gender, using data from the global burden of disease study 2017 (GBD 2017) [13].

## Methods

**Study design and data sources:** it was a descriptive study about DALYs trends by cause and gender from 1990 to 2017, in Tunisia. Due to the lack of exhaustive and recent data on death statistics in Tunisia, the source of data for this research was generated retrospectively from the global burden of diseases, injuries, and risk factors study (GBD) 2017 in accordance with the guidelines for accurate and transparent health estimates reporting [13].

To analyze DALYs trends, we extracted national estimates of numbers and age-standardized rate (ASR) of DALYs, years of life lost (YLLs) and years living with a disability (YLDs) with 95% uncertainty intervals (UIs), for every cause of death by NCDs, by sex and age group, from 1990 to 2017 in Tunisia using the GBD results tool [14,15].

**The global burden of diseases data base:** the GBD data base uses the disability-adjusted life-year (DALY) to measure the disease burden at the population level. DALYs are calculated by summing years of life lost (YLLs) due to premature mortality and years of life lived with disability (YLDs), thereby incorporating both fatal and non-fatal burden. YLLs were estimated using standard GBD methods whereby each death is multiplied by the normative standard life expectancy at each age. YLDs were estimated using sequelae prevalence and disability weights derived from population-based surveys [14,15]. All estimates generated in GBD were accompanied by 95% UIs, and age-specific death rate (ASDR) were estimated on the basis of the GBD world population age standard. According to the GBD data base, causes are classified into 4 levels. At level 1, there are three large cause groupings: communicable, maternal and neonatal conditions and nutritional deficiencies (CMNND); NCDs and injuries. At level 2 there are 21 disease and injury categories. The finest level of detail in causes is provided at levels 3 and 4. Causes reported within each level are mutually exclusive. Level 2 NCDs featured in GBD 2017 were: cardiovascular diseases (CVDs); neoplasms (cancers); chronic respiratory diseases; diabetes, urogenital, blood and endocrine disorders; neurological disorders; cirrhosis; digestive diseases; mental disorders; substance use disorders; musculoskeletal disorders; and other non-communicable diseases (including congenital anomalies, sense organ diseases, skin and subcutaneous diseases, and oral disorders) [13].

**Statistical analysis:** we used Joinpoint regression analysis to assess trends of the ASDR of NCDs from 1990 to 2017 and to determine average (mean) annual percentage change (APC) by a regressing log-linear function of the age-standardized DALYs per 100,000 population. APC was considered significant when it is different from zero at α=0.05. A constant trend was considered when the zero value was within both 95% UI limits for the APC, an increasing trend when both 95% UI limits were positive and a decreasing trend when both 95% UI limits were negative.

**Ethical considerations:** ethics approval was not required for this study.

## Results

**Figure 1:**
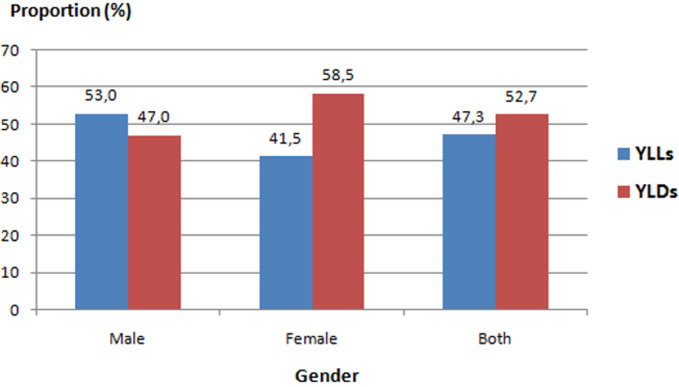
YLLs and YLDs proportion by gender among NCDs in 2017 (YLLs: years of life lost due to premature mortality; YLDs: years lived with disability)

**Figure 2:**
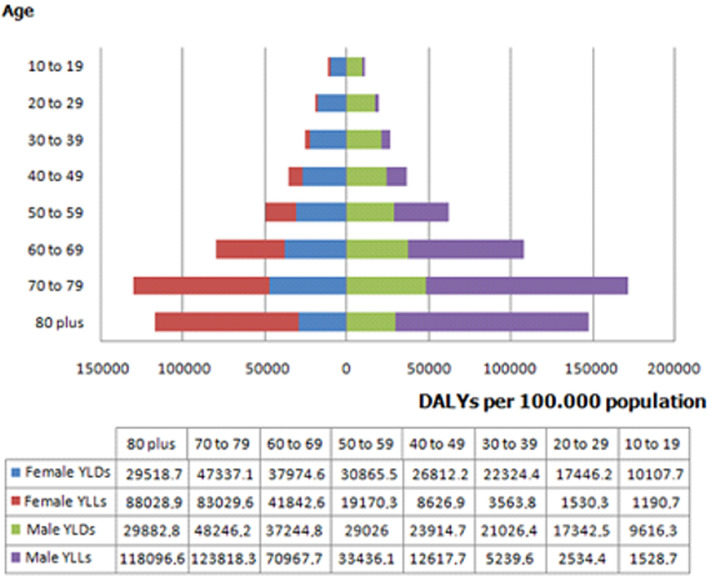
total disability-adjusted life years (DALYs) rates by gender and age group, Tunisia, 2017

**Burden of NCDs in Tunisia in 2017 by cause and gender:** NCDs accounted for the highest proportion of disease burden in Tunisia in 2017 representing 87.7% of total DALYs; while, this proportion was of 8.6% and 5.1% for CMNND and injuries respectively. Among total of DALYS for NCDs, 52.7% were from YLDs. Females contributed to a larger proportion of YLDs than males; however, the reverse was observed for YLLs (Figure 1). For both genders, NCDs were responsible of 18454.6 DALYs per 100.000 (15611.3 - 21555.4) in 2017. The distribution of ASDR by gender was higher in males with 20167.2 DALYs per 100.000 versus 16818.3 in females. CVDs were the leading level 2 causes of NCDs burden contributing 5102.5 per 100.000 DALYs (4297.7 - 6008.8) which represented more than the quarter of to¬tal ASDR (27.6%). CVDs were also the first leading cause groups of DALYs by gender, with 30.8% and 23.8% in males and females, respectively. The next most prominent causes were musculoskeletal disorders (MSD) (10.7%), neoplasms (10.0%), mental disorders (9.7%) and neurological disorders (8.7%) (Table 1). Comparison of genders, showed that the main difference of the most important level 2 cause groups of DALYs was for neoplasms; which were the second most common cause in males (11.1%) but the fifth ones in females (8.7%). A part from other NCDs, diabetes and kidney disorders and chronic respiratory diseases occupied the seventh and eighth place respectively (Table 1). According to the distribution analysis of DALYs by age groups, in both genders, the highest level of DALYs was observed in the 70-79 years age group, followed by 80 years and over, 60-69 years and 50-59 years age groups (Figure 2).

**Table 1:** ten leading broad cause groups of DALYs per 100.000 by gender, in Tunisia, 2017

	Cause groups DALYsper 100.000 [% of total DALYs]					
	Total		Male		Female	
	Non-communicable diseases	18454.6 (100%)	Non- communicable diseases	20167.2 (100%)	Non-communicable diseases	16818.4 (100%)
1	Cardiovascular diseases	5084.1 (27.5%)	Cardiovascular diseases	6217.6 (30.8%)	Cardiovascular diseases	4008.5 (23.8%)
2	Musculoskeletal disorders	1979.4 (10.7%)	Neoplasms	2234.8 (11.1%)	Musculoskeletal disorders	2035.1 (12.1%)
3	Neoplasms	1842.5 (10%)	Musculoskeletal disorders	1921.7 (9.5%)	Mental disorders	1943.1 (11.6%)
4	Mental disorders	1795.1 (9.7%)	Mental disorders	1638.3 (8.1%)	Neurological disorders	1861.9 (11.1%)
5	Neurological disorders	1607.5 (8.7%)	Neurological disorders	1343.5 (6.7%)	Neoplasms	1466.6 (8.7%)
6	Other non-communicable diseases	1300.1 (7%)	Other non-communicable diseases	1311.7 (6.5%)	Other non-communicable diseases	1276.8 (7.6%)
7	Diabetes and kidney diseases	1217.7 (6.6%)	Diabetes and kidney diseases	1228.3 (6.1%)	Diabetes and kidney diseases	1208.3 (7.2%)
8	Chronic respiratory diseases	859.5 (4.7%)	Substance use disorders	1182.4 (5.9%)	Sense organ diseases	808.8 (4.8%)
9	Sense organ diseases	799.7 (4.3%)	Chronic respiratory diseases	1046.5 (5.2%)	Chronic respiratory diseases	686.2 (4.1%)
10	Substance use disorders	760.3 (4.1%)	Sense organ diseases	790.1 (3.9%)	Digestive diseases	609.7 (3.6%)

**Trends of the ASDR of NCDs in Tunisia between 1990 and 2017:** regarding the trend of the ASDR due to NCDs for all ages, it decreased significantly from 23403.2 per 100.000 (20830.2 - 26285.8) in 1990 to 18454.6 (15611.3 - 21555.4) in 2017, with an APC of -0.9%; p=0.00. This trend was mainly observed for CVDs (essentially ischemic heart disease and stroke) with a significant APC of -1.4% (-1.8%; -1.0%); p=0.00. This decrease was observed at a lesser degree for neoplasms; the ASDR decreased from 1937.7 (1821.4 - 2047.9) to 1842.5 (1518.1 - 2205.1) with a non-significant APC of -0.3%. Mental disorders, neurological disorders, chronic respiratory diseases and sense organ diseases showed also a significant declining trends of the ASDR with an APC respectively of -0.01%, -0.1%, -0.8% and -0.7%; p=0.00.

On the other hand, the increase of the ASDR was due to MSD, diabetes, kidney disorders and substance use disorders with a significant APC of 0.1%, 0.2% and 1.3% (p=0.00) respectively during the 1990-2017 period, and also for some cancers including the overall ASDR of NCDs decreased in both genders with an APC of -0.7% in males and -1.1% in females (p=0.00). The decrease for CVDs (APC of -1.1% in males vs -1.9% in females; p=0.00) and neoplasms (APC of -0.4% in females; p=0.00) was more important in female in comparison to males; in fact, the ASDR of hypertensive heart disease and some other CVDs (such as atrial fibrillation and flutter, endocarditis and peripheral artery disease), which dropped among females, significantly increased in males. The decrease of the ASDR of neoplasms was mainly in females with a significant APC of -0.4%; p=0.00. In females, cervical cancer showed a declining trend. However, breast cancer and tracheal, bronchus and lung cancer were on the rise. Concerning colorectal cancers, a non-significant ascending trend among both genders was observed (Table 2, Table 2 (suite), Table 2 (suite 1), Table 2 (suite 2), Table 2 (suite 3), Table 2 (suite 4)). MSD, the second most common cause of DALYs rate in 2017 (Table 1), showed an increasing ASDR with a significant APC of 0.1% in both genders. Thus, the ASDR of diabetes raised also significantly but essentially in males with an APC of 0.5%. Substance use disorders, concerned particularly drug use disorders with an APC of 1.7% and 1.0% in males and females respectively (Table 2, Table 2 (suite), Table 2 (suite 1), Table 2 (suite 2), Table 2 (suite 3), Table 2 (suite 4)).

**Table 2:** trends of age-standardized DALYs by gender: 1990 to 2017

	Male			Female		
	1990	2017	Change	1990	2017	Change
NCD	24304.6 [27110.6-21658.8]	20167.2 [23721.9-16666]	-0.7^	22401.7 [25259.9-19810]	16818.4 [20210-13900.2]	-1.1^
Cardiovascular diseases	7658.4 [7084-8153.4]	6217.6 [4906.3-7609.4]	-1.0^	6435 [6097.3-6801.3]	4008.5 [3176.8-4921.6]	-1.9^
Rheumatic heart disease	58.9 [50.3-68.3]	18.2 [13.8-23.1]	-4.2^	80.1 [65.3-94.8]	19.8 [14.2-27.4]	-5.0^
Ischemic heart disease	5070.6 [4525.3-5570]	4097.7 [3197.1-5115.8]	-1.0^	3574.5 [3268.1-3963.2]	2152.6 [1633.3-2734.1]	-2.1^
Stroke	1827.9 [1617.1-2041.8]	1360.8 [1086.5-1692.5]	-1.2^	1895.2 [1695.6-2111]	1161 [920.3-1447.3]	-1.9^
Hypertensive heart disease	258.9 [133.9-325.6]	310.5 [105-447.5]	0.7^	477.5 [270.2-671.9]	354 [174.8-535.8]	-1.1^
Non-rheumatic valvular heart disease	28 [19.9-38.8]	27 [17.6-38.7]	-0.3	32.6 [27.2-38.8]	27.8 [20.2-37.6]	-0.7^
Cardiomyopathy and myocarditis	92.6 [73-113.6]	78.6 [50.1-103.1]	-0.7^	76.6 [58.6-103.4]	31.5 [24.1-41.7]	-3.2^
Atrial fibrillation and flutter	47.8 [36.5-61.5]	51.4 [39.7-66.4]	0.2^	55.8 [46.3-66.9]	57.8 [46.5-71]	0.1
Aortic aneurysm	24.7 [17.7-39.6]	27.9 [17.2-43.6]	0.2	8.4 [6.2-10.5]	8.3 [5.9-11.7]	-0.2
Peripheral artery disease	8.6 [4.5-14.6]	9.3 [5.3-15]	0.4^	7.6 [3.9-13.1]	7.7 [4.2-12.9]	0.1
Endocarditis	12.2 [9.3-20.2]	16.9 [11.2-22.8]	1.3^	14.1 [10.8-20.2]	10.9 [7.6-14.5]	-0.8^
Neoplasms	2243.6 [2049.3-2431.7]	2234.8 [1726.7-2803]	-0.2	1594.5 [1484.7-1714.9]	1466.6 [1115.3-1889.6]	-0.4^
Esophageal cancer	23.3 [20-26.8]	26.4 [19.6-34.4]	0.2	11.9 [10.2-14]	9.5 [7-12.4]	-1.0^
Liver cancer	44.3 [32.4-61.3]	51.9 [38.2-70.3]	0.5^	27.1 [21.8-34.2]	23 [16.4-30.9]	-0.8^
Larynx cancer	117.9 [100.3-142.1]	97.7 [72.4-126.8]	-0.9^	9.5 [8.2-10.9]	6.9 [5.1-9.1]	-1.3^
Tracheal, bronchus, and lung cancer	670.9 [582.1-767.4]	690.3 [518.1-893.2]	-0.2	61.4 [50.7-71.5]	78 [57.4-103.4]	0.8^
Breast cancer	4.7 [3.9-5.5]	5.1 [3.7-6.8]	0.0	323.6 [280.1-387.1]	378.5 [274.6-513]	0.2^
Colon and rectum cancer	137.3 [110.6-169.9]	165.9 [119.7-219.4]	0.4	151.3 [127-178.8]	145.8 [100.1-196.7]	-0.3
Lip and oral cavity cancer	38.9 [30.6-47.4]	38.8 [28.2-52]	-0.2	18.1 [15.8-20.7]	17.6 [12.9-23.5]	-0.2
Nasopharynx cancer	74.3 [64.1-88.6]	56.6 [41.5-75.1]	-1.3^	11.6 [9.7-13.6]	9 [6.5-12.3]	-1.0^

**Table 2(suite):** trends of age-standardized DALYs by gender: 1990 to 2017

	Male			Female		
	1990	2017	1990	2017	1990	2017
Other pharynx cancer	10.5 [7.3-13.5]	11.8 [7.8-16.7]	0.2	12.5 [10.3-15.2]	10.7 [7.4-14.5]	-0.8^
Gallbladder and biliary tract cancer	31.8 [22.6-39.4]	27.9 [18.2-38.7]	-0.6^	78.1 [58.8-93.2]	58.5 [40.5-79.4]	-1.2^
Pancreatic cancer	47.8 [39.6-57.9]	68.1 [48.5-89.4]	1.1^	32.4 [27.3-37.5]	48.7 [36-64.1]	1.4^
Malignant skin melanoma	7.8 [5.5-10.4]	8 [5.2-11.2]	-0.1	6.8 [4.5-9.7]	7.2 [10.4-4.5]	0.1
Non-melanoma skin cancer	10.1 [6.6-12.2]	11.3 [8.3-14.8]	0.5^	1.5 [1.2-1.7]	2.1 [1.5-2.7]	1.3^
Prostate cancer	103.6 [80.1-124.7]	119.5 [83-156.7]	0.5^	0	0	0
Testicular cancer	7.2 [5.5-8.9]	6.1 [4.3-8.4]	-0.9^	0	0	0
Cervical cancer	0	0	0	98.7 [81.2-117.6]	64.5 [47-86.7]	-1.8^
Uterine cancer	0	0	0	28.2 [24.4-32.8]	25.2 [18.3-34.2]	-0.6^
Ovarian cancer	0	0	0	58.3 [48.7-72]	72.7 [53.9-98.1]	0.6^
Kidney cancer	20.2 [15.3-24.6]	25.2 [18.2-33.5]	0.7^	14.1 [10.8-18.7]	13.5 [9.1-18.5]	-0.2^
Bladder cancer	115.8 [82.4-140.7]	127.9 [93.4-167]	0.3	17.0 [14.3-20.1]	16.7 [11.8-22.6]	-0.2
Brain and nervous system cancer	72 [48.9-102.3]	90.4 [64.1-118.7]	0.7^	81.3 [47.6-122.4]	56.2 [31.1-89]	-1.4^
Thyroid cancer	8.2 [6.6-9.9]	9.2 [6.7-12.2]	0.3	14.5 [12-19.6]	14.2 [9.9-20.3]	-0.2
Mesothelioma	1.4 [1-2]	4.5 [2.8-7.1]	4.4^	0.8 [0.6-1.1]	1.1 [0.7-1.5]	1.1^
Hodgkin lymphoma	39.6 [29-56.3]	25.1 [15-34.4]	-1.6^	38.4 [28.4-49.3]	21.5 [14.3-29.7]	-2.2^
Non-Hodgkin lymphoma	133 [105.2-157.2]	122.4 [89-160.9]	-0.4^	80.6 [68.8-91.8]	74.4 [55.7-98.8]	-0.4^
Multiple myeloma	24.8 [18.7-30.1]	28.9 [19.2-39.8]	0.4^	25.6 [20.8-30]	28.6 [19.5-38.6]	0.3^
Leukemia	174.4 [133.5-217]	143.4 [108.1-182.4]	-0.8^	138.8 [109.3-180.9]	90.1 [66.6-121.4]	-1.7^
Other malignant neoplasms	155.5 [131-186.7]	131.1 [99.9-165.7]	-0.7^	127.6 [110.2-147.9]	108.2 [81.7-142.5]	-0.7^
Other neoplasms	6.9 [4-12]	19.7 [12.9-34.7]	3.9^	8.7 [4.4-17.7]	14.1 [8.8-26]	1.8^

**Table 2(suite 1):** trends of age-standardized DALYs by gender: 1990 to 2017

	Male			Female		
	1990	2017	1990	2017	1990	2017
Chronic respiratory diseases	1220.8 [1074.8-1375.8]	1046.5 [872.9-1224.9]	-0.6^	855.9 [1000.4-731.7]	686.2 [824.6-574.2]	-0.8^
Chronic obstructive pulmonary disease	714.2 [603.8-817.3]	718 [603.3-840]	-0.0	456.2 [562.2-385.3]	382.4 [456.3-323.1]	-0.7^
Pneumoconiosis	3.4 [2.5-4.5]	3.4 [2.5-4.6]	-0.2	1.8 [3.4-0.8]	1.2 [1.7-0.7]	-1.6^
Asthma	468.3 [366.5-597.2]	268.8 [197.1-359.7]	-2.2^	368.6 [457.4-290.5]	258.2 [349.2-187.8]	-1.4^
Interstitial lung disease and pulmonary sarcoidosis	10.2 [6-22.2]	16.3 [9.9-30.1]	1.1^	8.8 [16.5-5.7]	11.8 [21.6-8]	1.1^
Other chronic respiratory diseases	24.7 [19.5-36.4]	40 [31.4-49.8]	2.0^	20.4 [25.3-16.3]	32.6 [40.5-25.5]	2.0^
Digestive diseases	1001.5 [1213.8-810.7]	755.5 [932.3-603.8]	-1.0^	857.3 [693.4-1052.3]	609.7 [480.9-777.8]	-1.2^
Cirrhosis and other chronic liver diseases	330.7 [435.2-240]	261.9 [351.3-192.7]	-1.0^	221.6 [174.1-282.8]	158.1 [119.9-206.3]	-1.3^
Upper digestive system diseases	373.7 [266.7-266.7]	264.8 [182.3-182.3]	-1.2^	400.3 [279-560.7]	285.8 [192.3-417.4]	-1.2^
Appendicitis	33.6 [42.3-20.6]	17 [23.3-10.5]	-2.7^	32.2 [24.3-41.7]	14.6 [10.5-19]	-3.0^
Paralyticileus and intestinal obstruction	63.4 [109.6-36.9]	32.4 [45.5-23.4]	-2.2^	55.2 [32.8-100.3]	23.9 [17-33.2]	-2.9^
Inguinal, femoral, and abdominal hernia	120.7 [164.2-81.4]	107 [148.7-70.8]	-0.4^	67 [45.5-92.5]	58.9 [39.6-81.1]	-0.4^
Inflammatory bowel disease	7.8 [11.4-5.4]	7.5 [10.2-5.4]	-0.1	8 [5.7-11.1]	8.7 [6-12.2]	0.4^
Vascular intestinal disorders	9.6 [14.3-6.6]	8.4 [12-6]	-0.5^	10.3 [7.4-15.5]	8.3 [5.7-14.3]	-0.9^
Gallbladder and biliary diseases	13.3 [18.5-9.4]	12.9 [18-9]	-0.1	16.7 [11.8-21.6]	14 [9-18.9]	-0.5^
Pancreatitis	14.2 [8.7-23.7]	13.9 [8.8-20.1]	-0.1	14.7 [10.4-18.8]	11.8 [8.1-15.9]	-0.8^
Other digestive diseases	34.4 [45.6-24.9]	29.6 [38.8-21.8]	-0.5	31.2 [23.5-40.4]	25.4 [19.2-32.6]	-0.7^

**Table 2(suite 2):** trends of age-standardized DALYs by gender: 1990 to 2017

	Male			Female		
	1990	2017	1990	2017	1990	2017
Neurological disorders	1353.7 [1725.2-1056.7]	1343.5 [1702.5-1035.9]	-0.0	1920.7 [1481.2-2443]	1861.9 [1399.2-2445.1]	-0.1^
Alzheimer's disease and other dementias	419.7 [494.5-337.3]	435.4 [532.4-346.1]	0.1	528.7 [469.4-581.5]	507 [406.8-621.2]	-0.2
Parkinson's disease	79.1 [105.2-65.2]	88.2 [114.2-68.1]	0.3^	54.9 [46.1-65.5]	53 [41.3-66.6]	-0.2
Epilepsy	156.2 [251.9-87.7]	115.9 [216.5-47.6]	-1.1^	153.8 [83-249.4]	106.7 [38.9-210.8]	-1.4^
Multiple sclerosis	9.5 [12.2-6.9]	11.7 [15.4-8.1]	0.6^	15.3 [11.1-19.6]	19.1 [13.7-25.3]	0.7^
Motor neuron disease	1.8 [3.1-1.2]	2.3 [5.1-1.5]	0.7^	1.3 [1-2.2]	1.7 [1.2-3.3]	0.9^
Headache disorders	659.2 [929.2-433.1]	657.9 [926.6-429.7]	-0.0	1138.7 [747.2-1601.5]	1145.2 [759-1613.8]	0.0^
Other neurological disorders	28.3 [45-16.4]	32.2 [54.4-15.9]	0.6^	28.1 [16.3-45.4]	29.2 [14.2-51.5]	0.2
Mental disorders	1658.6 [2145.5-1233.3]	1638.3 [2124.9-1213.4]	-0.1^	1960.9 [1447.4-2526.6]	1943.1 [1430.9-2514.3]	-0.0^
Schizophrenia	118.3 [149.2-87.4]	120.2 [150.6-87.6]	0.1^	115.8 [84.2-146.5]	118.1 [86.3-149.8]	0.1^
Depressive disorders	548 [749.2-383]	541.6 [737.3-380.6]	-0.0	776.7 [551.9-1066.8]	756.2 [530.2-1040.3]	-0.1^
Bipolar disorder	153.2 [225.9-94.5]	153.3 [229.4-95]	-0.0	169.7 [105.9-252.8]	170.4 [106.5-252.2]	0.0^
Anxiety disorders	341.1 [458.1-242.2]	341.5 [462.7-240]	-0.0	571.4 [408.7-760.6]	573.5 [407.5-765.3]	0.0^
Eating disorders	19.2 [28.6-11.8]	24.2 [36.1-15.1]	0.9^	44.1 [26.8-65.1]	55.9 [34.8-84.5]	0.9^
Autism spectrum disorders	75.1 [104.1-50.3]	75.3 [104.8-50.5]	0.0^	26 [17.4-36.2]	26.2 [17.6-36.5]	0.0
Attention/deficit/hyperactivity disorder	27.3 [43.4-16.3]	27.6 [44.5-16.6]	0.0^	11.5 [6.9-18.3]	11.6 [7-19]	0.0
Conduct disorder	124.8 [196.8-75.4]	125.1 [201-73.7]	0.0	69.9 [41.1-115.7]	70.1 [41.4-116.4]	0.0
Idiopathic developmental intellectual disability	84.3 [142.6-40.3]	62.2 [107-28.6]	-1.3^	63.5 [29.2-108.7]	48.3 [22.2-82.8]	-1.2^
Other mental disorders	167.4 [234.6-109]	167.3 [234.8-110.4]	-0.0	112.2 [74-155.4]	112.7 [74.9-156.2]	0.0^
Substance use disorders	772.7 [1003.9-549.7]	1182.4 [1531.7-879.4]	1.6^	286.9 [378-206.8]	355.3 [465.9-255.2]	0.8^
Alcohol use disorders	85.4 [116.2-59.7]	85.3 [116.7-59.8]	-0.1	53.5 [75.6-36.7]	54.4 [75.7-37.2]	0.0
Drug use disorders	687.3 [906-480.1]	1097.1 [1433.7-807.1]	1.7^	233.4 [315.6-163.8]	300.9 [398.7-213.2]	1.0^

**Table 2(suite 3):** trends of age-standardized DALYs by gender: 1990 to 2017

	Male			Female		
	1990	2017	1990	2017	1990	2017
Diabetes and kidney diseases	1058.7 [1241.4-898.7]	1228.3 [1495.2-965.6]	0.5^	1218.9 [1421.2-1045.5]	1208.3 [1486.1-953]	-0.1
Diabetes mellitus	622.3 [782-490.1]	852.1 [1086.8-648.1]	1.1^	723.9 [877.1-593.8]	837.8 [1060.5-632.2]	0.4^
Chronic kidney disease	433.8 [504.7-376.4]	375 [451.4-303.1]	-0.6^	494.3 [558-436.9]	369.7 [445.8-303.5]	-1.1^
Acute glomerulonephritis	2.6 [3.8-1.7]	1.2 [1.8-0.8]	-2.8^	0.6 [0.9-0.4]	0.7 [1-0.5]	1.1
Skin and subcutaneous diseases	468.4 [691.6-303.6]	496.8 [718.8-328.7]	0.2^	527.7 [773-343.5]	558 [802.9-370.6]	0.2^
Dermatitis	168.2 [95.5-273.2]	167.6 [94.4-272.5]	-0.0	199.6 [112.6-323.6]	199.4 [112.6-324.4]	-0.0
Psoriasis	44.4 [31-58.9]	60.5 [42.7-81.3]	1.2^	49.2 [34.6-64.8]	66.9 [47.2-89.3]	1.2^
Bacterial skin diseases	8 [4.2-19.4]	9.5 [5.2-24.4]	0.7^	7.4 [3.7-17.3]	8.8 [4.2-21.8]	0.6^
Scabies	34.6 [19-56.4]	30.6 [16.9-50.8]	-0.4^	36.3 [20.3-59.7]	32.1 [18-51.7]	-0.4^
Fungal skin diseases	38.3 [15.1-80.4]	35.7 [14.3-74.6]	-0.2^	36.2 [14.6-75.8]	34.1 [13.7-71.9]	-0.2^
Viral skin diseases	44.6 [28.2-66.6]	44.7 [28.4-67.3]	0.0	44.6 [28.3-66.8]	44.7 [28.1-67.3]	0.0^
Acnevulgaris	16.6 [9.8-27.1]	30.2 [17.8-48.4]	2.2^	17.4 [10.1-28.1]	30.6 [18.1-49.7]	2.1^
Alopeciaareata	6.2 [4-9.5]	6.2 [3.9-9.4]	0.0	6.2 [4-9.2]	6.2 [4-9.2]	0.0
Pruritus	8.3 [3.9-15.6]	8.6 [4.1-16.1]	0.1^	10.2 [4.7-19.4]	10.5 [4.9-19.8]	0.1^
Urticaria	63 [41.6-89.5]	63.1 [41.5-88.3]	0.0	82.8 [54.4-117.2]	83.2 [54.6-118.3]	0.0^
Decubitus ulcer	4.4 [3.1-6]	5.2 [3.6-7.1]	0.6^	4.4 [3.1-6.3]	4.9 [3.4-7.6]	0.4^
Other skin and subcutaneous diseases	31.9 [15.6-58.2]	34.9 [17-64.1]	0.3^	33.4 [16.2-61.1]	36.6 [17.8-67.2]	0.4^
Sense organ diseases	958 [667.8-1335.3]	790.1 [540.6-1110.5]	-0.7^	995.1 [697.7-1377.6]	808.8 [554.2-1135.8]	-0.7^
Blindness and vision impairment	556.5 [387.2-764.2]	396.5 [274.2-560.9]	-1.2^	627.4 [440-869.6]	445.6 [309.1-625.6]	-1.2^
Age-related and other hearing loss	373.8 [259.7-520.3]	364.9 [251.6-516.4]	-0.1^	335.6 [232-470.4]	330 [226.2-466.9]	0.0
Other sense organ diseases	27.7 [17-40.6]	28.7 [18-41.7]	0.1^	32.1 [19.8-46.9]	33.3 [20.5-48.8]	0.1^

**Table 2(suite 4):** trends of age-standardized DALYs by gender: 1990 to 2017

	Male			Female		
	1990	2017	1990	2017	1990	2017
Musculoskeletal disorders	1893.6 [1377.6-2470]	1921.7 [1399.9-2509.3]	0.1^	1983.3 [1436.2-2624.7]	2035.1 [1477.6-2694.3]	0.1^
Rheumatoid arthritis	14.1 [9.8-19.2]	16.3 [11-22.4]	0.6^	44.9 [31.3-60.6]	53.1 [36.4-70.9]	0.6^
Osteoarthritis	114.2 [56.8-227.1]	125.7 [63.5-248.3]	0.3^	124.7 [62.2-249.6]	138.2 [69.1-272.7]	0.4^
Low back pain	1038.2 [743.5-1363.4]	1016.1 [730.7-1344.8]	-0.1^	923.9 [648.5-1255.9]	919.2 [648.4-1246.6]	-0.0^
Neck pain	346.6 [242.5-485.9]	346.2 [240.6-482.4]	-0.0	531.1 [372.8-738.6]	533.5 [373.7-747.6]	0.0^
Gout	20 [13.6-27.8]	21.1 [14.3-29.4]	0.2^	6.5 [4.3-9.2]	7 [4.6-10.1]	0.3^
Other musculoskeletal disorders	360.6 [240.4-507.2]	396.2 [265.8-553.1]	0.3^	352 [240.4-488.9]	384.1 [263.2-527.7]	0.3^
Other non-communicable diseases	4016.5 [3207.2-4890.7]	1311.7 [1042.9-1625]	-3.9^	3765.6 [3168.6-4513.9]	1276.8 [994.4-1610.7]	-3.8^
Congenital birth defects	3280.5 [2507-4148.5]	768.2 [595-957.9]	-5.0^	2805.3 [2214.9-3484.1]	550.6 [435.2-678.6]	-5.6^
Urinary diseases and male infertility	373.7 [266.7-509.4]	264.8 [182.3-382.1]	0.1	13.7 [11.2-16.7]	14.3 [11.4-18]	0.2^
Gynecological diseases	0	0	0	282.6 [192.4-398]	276.8 [189.2-393.6]	-0.1^
Hemoglobinopathies and hemolyticanemias	120.7 [82.9-188.5]	51.3 [30.1-94.4]	-3.3^	193.3 [132-287.1]	76.8 [48.1-118.7]	-3.5^
Endocrine, metabolic, blood and immune disorders	71.8 [54.8-102.1]	54 [38-74.9]	-0.9^	107.1 [77.5-159.2]	60.6 [44-86]	-2.0^
Oral disorders	253.3 [155.2-388.9]	258.3 [159.4-393.4]	0.0	268 [165.2-406.7]	274.3 [169.5-414.5]	0.1^

^ p<0.05

## Discussion

The present study described the burden of NCDs in 2017 and analyzed DALYs trends by cause and gender in Tunisia during the 1990-2017 period, using data from the GBD [14]. According to this research, the burden of NCDs was very high; NCDs were responsible of 87.7% of the global ASDR; whereas only 8.6% were due to CMNND in 2017. The five leading level 2 causes of the ASDR of NCDs in Tunisia in 2017 were CVDs, MSD, neoplasms, mental and neurological disorders. Our findings have also shown that the trend of the ASDR of NCDs was significantly decreasing from 1990 to 2017, mainly for CVDs and at a lesser extent for neoplasms.

The declining trend of the burden of NCDs in Tunisia would be the result of treatment progress and a better management of patients, rather than the control of NCD´s risk factors. According to the WHO, more than 80% of NCDs can be prevented by eradicating the common risk factors, mainly tobacco use, unhealthy diets, physical inactivity and the harmful use of alcohol. Acting on modifiable causes of NCDs is the most cost-effective strategy to reduce the burden of NCDs and to improve longevity in the future [16]. In Tunisia, the prevalence of tobacco use remains high, despite the slightly decreasing trend during the period from 1990 (30%) to 2016 (25%) [17-19]; however, the prevalence of tobacco consumption was declining in almost all regions of the world [20]. Insufficient physical activity is one of the ten leading risk factors for global mortality due to NCDs. In Tunisia, more than the half (57.7%) of people aged over 15 years had insufficient physical activity [18]. The WHO mentioned that the East Mediterranean Region (EMR)(35%) had the highest prevalence of insufficient physical activity after the WHO region of America (39%) [21]. Food habits changes were the leading pattern of the life style shift, in line with the epidemiologic transition, WHO African and East Mediterranean Region (EMR) [22]. Principal dietary changes in Tunisia, between 1985 and 1995, were the reduction of the consumption of cereals, wheat, fish, fruits and vegetables against a rise intake of milk products, meat and fat [23]. In 2016, 80% of Tunisian aged over 15 years old did not consume enough fruits and vegetables per day [18]. Similar observations were described in the EMR, where dietary patterns consisted in low whole grains, low fruit, low vegetables and high sodium intake [24]. Obesity is also a real public health problem in Tunisia. In 2016, the average body mass index (BMI) in the population was of 26.9 ± 5.8 Kg/m^2^which mean an overweight [18]. In addition, the trend of the prevalence of obesity in Tunisia had increased from 10.9% in 1998 to 26.9% in 2016 [25-27].

To fight against NCDs and their risk factors in Tunisia, many strategies and programs were set up such as the program for the control of diabetes and hypertension, the cancer plan, the national strategy against CVDs, the national program for tobacco control, the national strategy for the prevention and control of obesity 2013 - 2017 etc. but, these actions were sparse and lacked multi-sectoral coordination due to a deep problem of governance and leadership [28]. Thus, the national strategy for the control and prevention of NCDs (including CVDs, cancers, chronic respiratory disorders and diabetes), based on WHO NCDs control strategy aimed to control main risk factors of NCDs by the reduction of the harmful use of chronic alcohol consumption by at least 10%, the reduction by 10% of sedentarily, the reduction by 30% of tobacco and salt intake, the reduction by 30% of hypertension and the stabilization of the prevalence of diabetes and obesity [12]. However, due to lack of budget, this strategy has not yet been implemented. Currently in Tunisia as well as all other countries, health policy makers focused mainly on the control of COVID-19 pandemic, however, this should not lead to loosening of NCDs control strategy. The decreasing trend of the ASDR of NCDs stated by this study is consistent with previous studies in high-income and some middle-income countries [29]. Yet, it is important to mention that the decreasing trend of the ASDR of CVDs was not observed diabetes and hypertensive heart disease essentially among males.

Neoplasms showed also a global declining ASDR; nevertheless, there were some differences between genders; the decrease was observed only in females. Cervical cancer showed a declining trend; which could be the results of primary prevention and in a less degree of the screening program. However, the burden of breast cancer was on the rise. Concerning colorectal cancers, the ASDR in both genders was steady. In fact, according to the national cancer plan in Tunisia for the 2015-2019 period, cancer screening is opportunist with a low the coverage [30]. Given the rising trend of breast cancer and secondly colorectal cancers in Tunisia, screening strategies need to be strengthened and the existing pilot screening projects need to be provided with sufficient material and human resources. A better awareness of the population and health care providers is recommended.

On the other hand, a part of the increasing trend of NCDs burden was related to MSD which occupied the second rank broad cause groups of DALYs in females and the third one in males, in Tunisia in 2017. Population growth, aging and sedentary lifestyles, would have contributed to this trend [31]. It is important to mention that MSD share risk factors common to other NCDs, such as obesity, unhealthy diet and a sedentary lifestyle [32]. To our knowledge, this is the first study in Tunisia assessing the burden trend of NCDs for al causes and by gender which is very important to alert health policy makers for the urgent need for action to control NCDs burden in Tunisia. The main limitation of this study was related to the use of estimated data from the GBD rather than national data. However, national data of causes of death are not valid; on the other hand, the quality GBD data four countries is satisfactory.

## Conclusion

Although the decreasing trend of NCDs burden in Tunisia from 1990 to 2017, mainly for CVD and neoplasms, this burden remains high. Reducing the levels of behavioral risk factors (tobacco use, physical inactivity, unhealthy diet) among Tunisian population are the key solutions to mitigate the impact of NCDs. A better governance for the implementation of the national strategy for the control and prevention of NCDs is urgently needed, underscoring the importance of leadership and the multisectoral coordination.

### What is known about this topic

- Non-communicable diseases (NCDs) are the leading cause of mortality worldwide;
- The sustainable development goals include a target of a one-third reduction in premature mortality from NCDs by 2030 relative to 2015 levels;
- In line with the WHO action plan, Tunisia carried out a national strategy for the prevention and control of NCDs for the 2018-2025 period.

### What this study adds

- This study describes and analyses global trends and changes of NCDs burden for al causes and by gender in Tunisia;
- This is very important to assess the achievement of the national strategy targeted goals and to alert health policy makers for the urgent need for action to control NCDs burden in Tunisia.
